# Prognostic Role of Chronic Rhinosinusitis in Acute Ischemic Stroke Patients Undergoing Mechanical Thrombectomy

**DOI:** 10.3390/jcm10194446

**Published:** 2021-09-27

**Authors:** Przemysław Puz, Grażyna Stryjewska-Makuch, Amadeusz Żak, Wiktor Rybicki, Sebastian Student, Anetta Lasek-Bal

**Affiliations:** 1 Department of Neurology, School of Health Sciences, Medical University of Silesia, ul. Ziołowa 45, 40-653 Katowice, Poland; azak@sum.edu.pl (A.Ż.); abal@sum.edu.pl (A.L.-B.); 2 Department of Neurology, Leszek Giec Upper-Silesian Medical Centre, Silesian Medical University, ul. Ziołowa 45, 40-653 Katowice, Poland; wmrybicki@gmail.com; 3 Department of Laryngology and Laryngological Oncology, Leszek Giec Upper-Silesian Medical Centre, Silesian Medical University, ul. Ziołowa 45, 40-653 Katowice, Poland; makuch_mg@wp.pl; 4 Biotechnology Center, Faculty of Automatic Control, Electronics and Computer Science, Silesian University of Technology, ul. Akademicka 16, 44-100 Gliwice, Poland; Sebastian.Student@polsl.pl

**Keywords:** stroke, mechanical thrombectomy, chronic rhinosinusitis, prognosis

## Abstract

Background: The aim of the study was to assess the relevance of chronic rhinosinusitis (CRS) CT features to the efficacy of mechanical thrombectomy (MT) in patients with acute ischemic stroke. Methods: This study included 311 patients qualified for MT in whom the CRS features were assessed based on a CT scan, according to the Lund-Mackay (L-M) score. Clinical, inflammatory parameters, patients neurological (NIHSS) and functional status (mRS), and recanalisation efficacy (TICI) were compared between patients with mild lesions (L-M score 0–3 points)-group 1, and patients with more severe lesions (L–M score 4–24)-group 2. Results: There was a significant difference in the NIHSS on day seven after stroke onset-10 points in group 1 and 14 points in group 2, *p* = 0.02. NIHSS ≤ 6 points on day seven was found in 41.9% of patients in group 1, and in 27.5% in group 2, *p* = 0.042. There were no significant differences in mRS score and in the TICI score. L-M score, lipid abnormalities and CRP were factors associated with NIHSS ≥ 7 points on day 7. Conclusions: The CT features of CRS may be used as a prognostic tool for early prognosis assessment in stroke patients.

## 1. Introduction

Endovascular treatment of ischemic stroke patients is now a standard therapy for the acute phase of the disease. The efficacy and safety of mechanical thrombectomy has been validated in several randomized clinical trials [1,2,3,4]. Based on the results of the first meta-analysis of the HERMES data gathered in five randomized trials, the use of mechanical thrombectomy results in the recanalization of more than 80% of arteries subjected to intervention and in the return to full independence within three months after stroke in 46% of patients [5]. However, this means that a significant neurological post-stroke deficit remains in half of the patients, including some of those who had favorable angiographic results after mechanical thrombectomy. Factors associated with the efficacy and safety of the endovascular procedures as well as factors determining the clinical outcome in patients with acute stroke undergoing mechanical thrombectomy are still being sought.

The unfavorable prognosis parameters identified in the subpopulation of patients treated by endovascular therapy due to stroke include: old age, diabetes mellitus, admission stroke severity, poor neurological status on the first day of stroke, and low Alberta Stroke Program Early CT Score (ASPECTS) [6,7,8,9].

Some of the potential factors determining the prognosis in stroke patients after thrombectomy also include chronic inflammation. Chronic rhinosinusitis (CRS) is one of the most common inflammatory conditions. CRS has been associated with an increased risk of cardiovascular events, including ischemic stroke [10,11,12,13].

The anatomical location of CRS, its immediate proximity to the central nervous system (CNS), and the fact that previous data on the prognostic relevance of CRS in patients undergoing thrombectomy and on the prognostic relevance of inflammatory process in stroke patients is insufficient; it has been suggested that further research in this area is needed.

Determining the impact of CRS on the prognosis in patients after mechanical thrombectomy seems to be of great importance. The presence of larger inflammatory foci in the immediate vicinity of vessels can be important not only due to continuous infectious spread but also to the secretion of numerous inflammatory mediators in the Th1, Th2, and Th17 cell line affecting the intima-media complex [14,15].

The aim of this study was to assess the relevance of the extent of inflammatory lesions within the paranasal sinuses, as visualized in imaging, to the radiological and clinical efficacy of endovascular thrombectomy in patients with acute ischemic stroke.

## 2. Materials and Methods

This retrospective study included patients referred to the Department of Neurology of the Upper-Silesian Medical Centre in Katowice Ochojec from 1 January 2019 to 31 August 2020 with ischemic stroke indicated for mechanical thrombectomy in accordance with the 2018 Guidelines for Management of Acute Ischemic Stroke [16]. The presence of embolic material in the large cerebral vessels in all patients was confirmed by CT angiography.

All patients were assessed and qualified for thrombectomy by the stroke team: the neurologist, the anesthetist, and the operator of the procedure. We included patients with functional independence before the occurrence of stroke (pre-stroke 0–2 on the modified Rankin scale, mRS). No age limit was applied to adult patients. Exclusion criteria were the following: failure to meet procedure time limits (6 h from symptoms onset), pre-stroke disability (mRS > 2 points), occlusion in distal cerebral arteries, intracerebral hemorrhage on imaging or clinical signs of subarachnoid hemorrhage (revealed in non-contrast computed tomography), uncontrolled arterial hypertension (systolic blood pressure > 180 mmHg or diastolic blood pressure > 110 mmHg, immediately before endovascular treatment), progressive damage to the central nervous system other than acute stroke (e.g., cancer, previous surgery with cranial or spinal canal opening), cirrhosis or portal hypertension, and pregnancy. Mechanical thrombectomy procedures were performed in the Comprehensive Stroke Center equipped with a state of the art machine, the Siemens Axiom Artis Zee Bi-Plane. Most of the mechanical thrombectomy procedures were done with the use of stent retrievers (Trevo, Catch, Soliter, Tiger) and the 8F Balloon Guide Catheter (Flow Gate 2). For the remaining material distal aspiration (Penumbra, Sofia, Cathalist) or combined stent retrievers and distal aspiration is used. General anesthesia was available and used when necessary. Neurosurgical protection was warranted at the site. Periprocedural anticoagulation with heparin 50–70 IU/kg was used. After the procedure, patients received standard stroke therapy (acetylsalicylic acid 150 mg or clopidogrel 75 mg or anticoagulation if necessary).

All patients were rated in relation to the occurrence of stroke risk factors and comorbidities, which included: atrial fibrillation (AF), coronary artery disease (CAD), mitral and/or aortic valve stenosis, the presence of a mechanical heart valve, previous myocardial infarction, arterial hypertension (AH), diabetes mellitus (DM), high body mass index (BMI) > 25 kg/m^2^, abdominal aorta atherosclerosis (AAA), lower extremity arterial disease (LEAD), lipid disorders, nicotinism, and positive family history. All of the patients were also rated in relation to the presence of clinical or biochemical signs of inflammation (respiratory tract infection, urinary tract infection, gastritis, C-reactive protein (CRP) plasma concentration and white blood cell (WBC) count).

The recanalisation efficacy of the endovascular treatment applied was assessed according to the Thrombolysis In Cerebral Infarction (TICI) classification [17]. Patients with a TICI score of 2b–3 were classified as those who underwent successful thrombectomy. The patients’ neurological status was assessed using the National Institutes of Health Stroke Scale (NIHSS) [18]. For the purposes of this study, the NIHSS neurological status was assessed at the time of onset and seven days after the onset, and a change in NIHSS scores (improvement of at least 4 points) on day seven as compared to the day of onset was considered.

The functional status was assessed using the mRS on the seventh day after the procedure [19].

In both groups, the authors assessed the extent of the inflammatory process in the paranasal sinuses based on a CT scan, without contrast enhancement, performed on the first day of hospitalization, using an Optima CT 540.

The sinuses were assessed in three planes. The inflammatory lesions were assessed according to the Lund-Mackay score (L-M) (the degree of opacification in the maxillary, anterior and posterior ethmoid, frontal, and sphenoid sinuses as well as the obstruction of the ostiomeatal complexes were evaluated (on both sides) on a 0–2 scale-a maximum of 24 points) [20]. Depending on the number of points, the following categories of CRS severity were distinguished: 0-nonrmal, 1 to 3-mild, 4 to 10-moderate, >10 severe. CRS severity grading was performed by two (or three when needed) experienced specialists, who agreed with the assessment of the Lund-Mackay score. The authors use the term CRS conventionally considering only sinus inflammatory changes visible in the head CT scan, without data on clinical complaints and treatment, as suggested by European Position Paper on Rhinosinusitis and Nasal Polyps (EPOS) [20,21]. Patients with CT images insufficient for the assessment of sinuses were excluded from further analysis.

Patients with a normal sinus picture or with mild lesions as shown with the scores L-M (0–3 points) were classified as Patient Group 1, while patients with moderate to severe lesions (L–M score 4–24) were classified as Patient Group 2.

Groups 1 and 2 were compared in terms of their clinical features, inflammatory parameters, the neurological status (according to NIHSS) on days one and seven, improvement in the neurological status, the functional status of patients according to their mRS score on day seven, and recanalisation efficacy according to TICI classification (TICI 0–2a vs. 2b–3).

For the purpose of this study, NIHSS ≥ 7 pts assessed seven days after symptoms onset was assumed as an unfavorable clinical status. We searched for independent demographical, clinical, biochemical and imaging factors associated with NIHSS ≥ 7 pts on day seven, including the L-M score.

## 3. Statistical Analysis

Statistical analysis was performed with the tests set out below. Basic statistical parameters were calculated for interval scale variables (mean, standard deviation (SD), median, and range). Compliance of the distribution of these variables with normal distribution was verified using the Shapiro-Wilk test. Count and percentage distributions in respect of variable categories were set for nominal variables. Comparisons of mean/median values in the case of interval scale variables were made using the *t*-test (for variables with normal distribution) or the Mann–Whitney U test (for variables where the distribution differed from normal distribution). Comparisons of groups in the case of nominal variables were performed using the chi-squared test or Fisher’s test, depending on the size of the groups. The comparison of mean change of NIHSS score from day one to day seven between Group 1 and 2 were calculated by using the repeated measures ANOVA test. In order to identify risk factors for unfavorable clinical status–NIHSS ≥ 7 pts was assessed seven days after symptom onset, and ordinal regression was applied to the data using R environment (ver. 3.6.1). The model’s variable selection procedures included automatic selection (forward stepwise search) based on the Akaike information criterion (AIC). The model meets the requirements of linearity, is well accommodated to the count rates data, and the data are distributed evenly. For evaluating the accuracy of the predictions made by the model, the “leave one out” procedure and the area under the ROC curves (AUC) were used. It included WBC, CRP, AH, DM, age, sex, lipid disorders, AF, LEAD, smoking, internal carotid artery stenosis >50%, and L-M score. A significance level of *p* ≤ 0.05 was assumed to be substantial.

Analyses were performed using Statistica software (version 13.3; Tibco Software Inc., Palo Alto, CA, USA).

The study was not a medical experiment and, as such, was not required to be evaluated by the Bioethics Committee of the Medical University of Silesia in Katowice.

## 4. Results

In the period between 1 January 2019 and 31 August 2020, a total of 311 individuals underwent thrombectomy. The extent of inflammatory lesions in the paranasal sinuses was assessed in all of these patients; 255 patients were eventually qualified for evaluation after excluding those with insufficient CRS records.

Normal sinus pictures or radiological signs of sinusitis visualized in head CTs of mild severity (0–3 points for CRS) were found in 186 patients (Group 1), whereas the signs of moderate to significant severity were recognised in 69 patients (Group 2). The clinical description of patients including stroke risk factors is presented in Table 1. There were no statistically significant differences between Patient Groups 1 and 2.

There were no differences in the examined inflammatory parameters (WBC and CRP) between the group of patients with mild inflammation and the group of patients with moderate to advanced sinusitis. In Group 1, the mean CRP concentration was 18.45 ± 24.91 mg/L; in Group 2, the mean CRP concentration was 19.16 ± 20.14 mg/L (*p* = 0.46). the mean WBC count in Group 1 was 10.79 ± 3.87 thousand per ml and in Group 2 it was 11.35 ± 4.58 thousand per ml (*p* = 0.65). The clinical evaluation did not reveal any differences in the prevalence of symptoms of acute infection between the study groups. Clinical symptoms of acute infection (respiratory or urinary tract infection) were found in 21 (11.3%) patients in Group 1 and in nine (13%) patients in Group 2, *p* = 0.67.

Intravenous thrombolytic therapy (iv rtPa) was given to 124 patients (66.7%) in Group 1 and 51 patients (73.9%) in Group 2 (*p* = 0.29). Moderate to severe CRS signs (L–M score 4–24) were found in 51 (29.1%) patients in whom iv rtPa, together with endovascular treatment was applied, and in 18 (22.5%) patients without iv rtPa, the difference was not statistically significant, *p* = 0.29.

No significant differences were found between the study groups in terms of the patients’ clinical status as assessed using the NIHSS at admission (day one): the NIHSS median for Group 1 was 11 (range 1–30, mean 11.5 ± 5.08), and for Group 2 it was 12 (range 3–26, mean 13.2 ± 6.59), *p* = 0.14.

There was a significant difference in terms of the patients’ clinical status as assessed using the NIHSS on the seventh day after the procedure; for Group 1, the NIHSS median on day seven post-procedure was 10 points (range 0–27 mean 10.1 ± 5.52), while for Group 2 it was 14 points (range 0–26, mean 12.9 ± 6.59), *p* = 0.02. The mean change of NIHSS score from day one to day seven was 1.42 ± 1.33 in Group 1 and 0.43 ± 0.66 in Group 2, *p* = 0.37.

In Group 1, the neurological status assessed using the NIHSS at ≤6 points on day seven after the onset was found in 78 patients (41.9%), and in Group 2 it was found in 19 patients (27.5%), *p* = 0.042.

Improved neurological status as assessed using the NIHSS for the aforementioned four points was observed in 49 patients (26.3%) in Group 1 and in 11 patients (15.9%) in Group 2; however, that difference failed to be statistically significant, *p* = 0.13.

There were no significant differences in the functional status as assessed using the mRS on day seven after onset; the mRS median in Group 1 was 4 points (range 0–6) and in Group 2 the mRS median was 5 points (range 0–6), *p* = 0.14. The number of patients with good functional status (mRS < 3) on day seven after thrombectomy in Group 1 was 55 (29.6%) and in Group 2 it was 12 (17.4%), *p* = 0.15.

There was no difference between the percentage of patients in the two groups who achieved a complete and successful recanalisation of the intracranial artery (TICI2b–3): 119 (64.0%) in Group 1 vs. 43 (62.3%) in Group 2, respectively; *p* = 0.81.

Comparisons of clinical and radiological outcomes after endovascular treatment between Patient Group 1 and Patient Group 2 are shown in Table 2.

Risk factors of unfavorable clinical outcome (NIHSS ≥ 7 pts on day 7) were found in an ordinal regression model related to age, sex, stroke risk factors, the inflammatory condition parameters studied, and the L-M score. Lipid abnormalities, CRP and the L-M score were concluded to be independent factors associated with the patients’ clinical status as assessed using the NIHSS score ≥ 7 points on day seven following the procedure (Table 3). The AUC, sensitivity, and specificity, for the model was 0.672, 0.45, and 0.92, respectively.

## 5. Discussion

The main finding of this single-centre retrospective observational case-control study was the confirmation of the relevance of inflammatory lesions in the paranasal sinuses as evaluated with head CT to early clinical prognosis in patients after a recent stroke. The neurological status assessed using the NIHSS 7 days after thrombectomy in patients with mild signs of chronic sinusitis was significantly better than in those with moderate to advanced sinusitis. CRS assessed with head CT upon admission was also found to be an independent factor influencing the NIHSS score in our ordinal regression analysis considering the potential factors influencing the clinical status of patients in the acute phase of ischaemic stroke.

A significant clinical improvement (NIHSS scores lowered by at least 4 points) was observed in 26.3% of the patients with mild CRS and in 15.9% of the patients with moderate to advanced sinusitis. However, that difference was not statistically significant. The lack of any statistical significance of the comparison performed could be justified by large differences between study group sizes (186 patients in Group 1 and 69 patients in Group 2). The small size of Group 2, including patients with moderate (42 patients) and advanced (27 patients) sinusitis, encouraged us to combine these into a single group so as to avoid comparing such groups that are significantly heterogeneous in terms of size.

The relevant literature reports multiple examples of clinical, biological, biochemical, and radiological factors with potential relevance to the prognosis for ischaemic stroke patients treated with mechanical thrombectomy [22,23]. The factors associated with a poorer prognosis following thrombectomy include: age, comorbitidies (HA, DM), previous stroke, stroke severity, the Alberta Stroke Program Early CT Score (ASPECTS), ischemic core volume, mismatch of ischemic core and hypoperfusion volume, white matter lesions, the location of the occlusion, collateral flow status and neutrophil lymphocyte ratio [5,6,7,8,24,25].

To our knowledge, this is the first study assessing the impact of CRS on the short-term prognosis in patients with stroke. There are reports in the literature claiming that patients with chronic inflammatory diseases have a higher risk of coronary heart disease, peripheral vascular disease, cardiomyopathy and stroke. Inflammation also promotes the development of arteriosclerosis. Chronic rhinosinusitis (CRS) is one of the most widespread chronic diseases in the world, affecting 11% of Europe’s population and 12.5% of the US population [26,27,28]. There are known complications of CRS resulting from the anatomical proximity of the sinuses and the brain, such as brain abscesses, subdural empyema, cranial nerve palsy and meningitis. Chronic rhinosinusitis can lead to cavernous sinus, jugular vein thrombosis, and mycotic aneurysm [29,30,31]. There is also a relationship between chronic inflammatory diseases and cardio-cerebro-vascular diseases (CCVDs) [32]. Our previous study, as well as other cohort studies, revealed that patients with CRS were at a higher risk of stroke or acute myocardial infarction [10,11,12,33].

In the present study, no relationship was found between CRS and TICI. In the available literature, there is no data related to any significant association between CRS or any chronic inflammatory condition and the efficacy of recanalisation in mechanical thrombectomy. The factors associated with poorer rates of successful recanalisation and early reocclusion following mechanical thrombectomy include old age, moderate and severe stroke, internal carotid artery occlusion, distal vascular embolism, intravenous thrombolysis, surgical and instrument-related complications, delayed opening of blood vessels, number of thrombectomy passes, stent implantation, and levels of D-dimer [34,35].

We found no differences in the functional status of patients between the group with mild inflammatory lesions of the sinuses and the group with moderate to advanced sinusitis as assessed using the mRS on day seven following onset. A seven-day follow-up period is too short for us to assess significant improvements in the patients’ functional status using the mRS. All clinical trials, including multicentre randomised trials focusing on new therapies for stroke, apply the mRS functional status score assessed over a three-month period following mechanical thrombectomy.

From the clinical point of view, our results justify particular attention to the paranasal sinuses in patients with stroke qualified to the mechanical thrombectomy. When moderate or severe CRS is diagnosed in these patients, intensive maximal medical therapy or interventional treatment should be started as early as possible, as this may improve short-term efficacy of endovascular treatment of patients with stroke.

## 6. Limitations of the Study

- the retrospective nature of the study: the ability to design a prospective analysis which would enable us to assess the relevance of CRS in stroke patients treated with mechanical thrombectomy was quite limited;
- insufficient clinical follow-up period (seven days): a standard assessment of the patients’ functional status using the mRS at three months following the onset and thrombectomy was not the subject of the present study. During a three-month period after onset, various methods of therapeutic management and rehabilitation were employed in the study group of patients who presented additional diverse conditions, and this resulted in high heterogeneity of the study group and a possible bias related to the three-month follow-up;
- the lack of data from the history of sinus complaints and possible treatment in accordance with the EPOS 2020 guidelines [20,21]. There were no results of subjective tests, such as the visual analogue scale or the Sino-Nasal Outcome Test-22, or accurate data on inhalation allergy, bronchial asthma and hypersensitivity to non-steroidal anti-inflammatory drugs (NSAIDs).

A possible limitation, yet an advantage in the studies presented here, is the application of head CT to assess the inflammatory lesions of the sinuses. In the acute phase of stroke and around the time of thrombectomy performance, any diagnostic procedures which could delay thrombectomy should be limited; therefore, performing an imaging investigation targeted on the sinuses is not feasible in this specific period. On the other hand, the ability to assess CRS on the basis of head CT performed upon admission makes it possible for us to use another diagnostic tool for the evaluation of early prognosis in patients with ischaemic stroke. Sinus inflammatory changes detected by CT of the head were objective confirmation of chronic inflammation of varying severity, which could not be related to hospital-acquired infection due to the fact that it was performed on the first day of hospitalization. No changes in the sinuses suggesting cancerous tumours (bone infiltration and destruction or spread beyond the sinuses) were found.

## 7. Conclusions

The computed tomography features of CRS revealed in patients with acute stroke undergoing mechanical thrombectomy are connected with worse clinical outcomes.

The presence of CRS may be used as a prognostic tool for the early prognosis assessment in stroke patients.

The findings of this study should be interpreted within the context of the retrospective study design and sample size.

## Figures and Tables

**Table 1:** Clinical characteristics of patients.

Parameter	Group I (CRS 0–3), *n* = 186	Group II (CRS 4–22), *n* = 69	*p*-Level
Age, mean SD median min–max	69.81 12.66 72 25–92	66.75 11.22 67 40–87	0.14
Sex, female (*n*, %)	98, 52.7	27, 39.1	0.15
AF (*n*, %)	91, 48.9	40, 57.9	0.59
AH (*n*, %)	137, 73.7	54, 78.3	0.96
DM (*n*, %)	53, 28.4	15, 21.7	0.96
LEAD (*n*, %)	74, 39.7	30, 43.5	0.96
Smoking (*n*, %)	18, 9.7	11, 15.9	0.44
LD (*n*, %)	104, 55.9	37, 53.6	0.59
ICA stenosis > 50% (*n*, %)	67, 36.0	32, 46.4	0.15

SD—standard deviation, AF—atrial fibrillation, AH—arterial hypertension, DM—diabetes mellitus, LEAD—lower extremity arterial disease, LD—lipid disorders, ICA—internal carotid artery.

**Table 2:** Comparison of clinical and radiological outcomes after endovascular treatment between Patient Group 1 and Patient Group 2.

Outcome	Patient Group 1, *n* = 186	Patient Group 2, *n* = 69	*p*
NIHSS day 7			
median, range	10 (0–27)	14 (0–26)	0.02
mean ± SD	10.1 ± 5.52	12.9 ± 6.59	
NIHSS ≤ 6 pts on day 7			
*n*, %	78 (41.9%)	19 (27.5%)	0.042
NIHHS improvement to day 1 > 4 points			
*n*, %	49 (26.3%)	11 (15.9%)	0.13
mRS day 7			
median, range	4 (0–6)	5 (0–6)	0.4
mRS < 3 points on day 7			
*n*, %	55 (29.6%)	12 (17.4%)	0.15
TICI 2b-3			
*n*, %	119 (64%)	43 (62.3%)	0.81

NIHSS—National Institutes of Health Stroke Scale, mRS—modified Rankin Scale, TICI—Thrombolysis in Cerebral Infarction.

**Table 3:** The results of logistic regression analysis: the factors affecting the patients’ clinical status (NIHSS ≥ 7 points) on day seven after thrombectomy.

Coefficients	OR	CI 95%	*p*-Value
WBC (>10 × 3/mL)	1.159	(0.864–1.562)	0.321
CRP (>5 mg/L)	1.021	(1.007–1.044)	0.047
AH	0.582	(0.065–4.935)	0.621
DM	0.607	(0.096–3.5)	0.579
Age per year	1.05	(0.973–1.137)	0.214
Sex female	0.677	(0.137–3.168)	0.62
LD	1.114	(1.019–1.531)	0.009
AF	0.518	(0.095–2.663)	0.433
LEAD	1.486	(0.241–9.526)	0.668
Smoking	0.634	(0.094–4.26)	0.635
ICA stenosis > 50%	0.321	(0.024–3.638)	0.361
L-M score (per point)	1.077	(1.001–1.164)	0.049

OR—odds ratio, CI—confidence interval, WBC—white blood cell count, CRP—C-reactive protein, AH—arterial hypertension, DM—diabetes mellitus, LD—lipid disorders, AF—atrial fibrillation, LEAD—lower extremity arterial disease, ICA—internal carotid artery, L-M—Lund-Mackay score.

## Data Availability

The data presented in this study are available on request from the corresponding author. The data are not publicly available due to privacy restrictions.
